# Cellular Immune Response against Nontypeable *Haemophilus influenzae* Infecting the Preinflamed Middle Ear of the *Junbo* Mouse

**DOI:** 10.1128/IAI.00689-19

**Published:** 2019-11-18

**Authors:** Pratik P. Vikhe, Tom Purnell, Steve D. M. Brown, Derek W. Hood

**Affiliations:** a Mammalian Genetics Unit, MRC Harwell Institute, Oxfordshire, United Kingdom; University of California San Diego School of Medicine

**Keywords:** nontypeable *Haemophilus influenzae*, acute otitis media

## Abstract

Nontypeable Haemophilus influenzae (NTHi) is a major pathogen causing acute otitis media (AOM). The pathology of AOM increases during long-term infection in the middle ear (ME), but the host cellular immune response to bacterial infection in this inflamed environment is poorly understood. Using the *Junbo* mouse, a characterized NTHi infection model, we analyzed the cellular response to NTHi infection in the *Junbo* mouse middle ear fluid (MEF). NTHi infection increased the total cell number and significantly decreased the proportion of live cells in the MEF at day 1, and this further decreased gradually on each day up to day 7. Flow cytometry analysis showed that neutrophils were the dominant immune cell population in the MEF and that NTHi infection significantly increased their proportion whereas it decreased the monocyte, macrophage, and dendritic cell proportions. Neutrophil and macrophage numbers increased in blood and spleen after NTHi infection. The T-cell population was dominated by T-helper (Th) cells in noninoculated MEF, and the effector Th (CD44^+^) cell population increased at day 2 of NTHi infection with an increase in IL-12p40 levels. Sustained NTHi infection up to 3 days increased the transforming growth factor β levels, decreasing the effector cell population and increasing the T-regulatory (T-reg) cell population. In the preinflamed ME environment of the *Junbo* mouse, neutrophils are the first responder to NTHi infection followed by T-reg immune suppressive cells. These data indicate that sustained NTHi infection in the ME induces the immune suppressive response by inducing the T-reg cell population and reducing immune cell infiltration, thus promoting longer-term infection.

## Abstract (precis)

Nontypeable Haemophilus influenzae (NTHi) is a major pathogen causing acute otitis media (AOM). The pathology of AOM increases during long-term infection in the middle ear (ME), but the host cellular immune response to bacterial infection in this inflamed environment is poorly understood. Using the *Junbo* mouse, a characterized NTHi infection model, we analyzed the cellular response to NTHi infection in the *Junbo* mouse middle ear fluid (MEF).

## INTRODUCTION

Otitis media (OM) is characterized by inflammation and accumulation of fluid in the middle ear (1). Acute OM (AOM) is a common form that is initiated by bacterial infection; the pathology of AOM increases with the long-term infection typically associated with the common otopathogens nontypeable Haemophilus influenzae (NTHi), Moraxella catarrhalis, and Streptococcus pneumoniae (2, 3). Due to lack of a vaccine, record numbers of children from both the developing and developed world suffer from recurrent OM caused by NTHi infection (2). The interesting factor regarding these infections is that the pathogens can survive in the middle ear fluid (MEF) that results from inflammation and thus consists of immune cells (4, 5) and many molecules with potential antibacterial activity (6). NTHi must have evolved strategies (7–9) to survive in the inflamed middle ear, but many of the details of how NTHi manipulates the immune environment in the inflamed middle ear to ensure its long-term survival remain unclear.

Immune-competent cells infiltrate the middle ear following infection and inflammation (10). Inoculation of the pneumococcus into the chinchilla middle ear induces interleukin-1β (IL-1β) secretion, followed by IL-6, IL-8, and tumor necrosis factor alpha (TNF-α) and neutrophil infiltration into the middle ear (11). In human, another innate immune cell, the dendritic cell (DC), shows some difference in OM-prone compared to nonprone children. Dendritic cells isolated from OM-prone children show lower major histocompatibility complex class II (MHC-II) expression on their surfaces (4), indicating that they are less able to induce a T-cell maturation response. Also, natural killer cells increase in the blood of children with chronic suppurative OM (CSOM), suggesting a possible role of these cells in middle ear infection (12, 13). In the rat model of AOM, induced by severing the soft palate, local proliferation of macrophages, dendritic cells, natural killer (NK) cells, and T and B lymphocytes was observed on day 5 postinoculation (14), suggesting an involvement of the local lymphatic system in the middle ear cellular and inflammatory response.

The adaptive immune response in children prone to AOM has been investigated to understand the lack of immune clearance of NTHi infection. Patients that are more susceptible to NTHi infection exhibit a reduced memory-dependent response and are inclined to have a Th2-dependent immune response (4). Sustained NTHi infection and inflammation in the mouse middle ear following direct middle ear NTHi inoculation after blocking the Eustachian tube induce T-regulatory (T-reg) cell-mediated immune suppression, thus contributing to induction of tolerance against NTHi (15), and may be a critical factor in lack of a memory-dependent immune response.

All of the animal models used to investigate the middle ear cellular and inflammatory response against NTHi infection do so by direct inoculation into the middle ear of the animal with or without prior alteration of the Eustachian tube. In contrast, in our study we use the *Junbo* mouse, a mutant mouse line that is a well-characterized chronic OM with effusion (COME) (16) and AOM (17) model. The *Junbo* mouse spontaneously generates middle ear inflammation and accumulation of the middle ear fluid at around 4 to 5 weeks of age (16). Inoculation via the nasal passage, which is a natural route of NTHi infection, results in significant and sustained NTHi infection in the middle ear of the *Junbo* mouse (17). In this infection model, middle ear fluid provides a natural *in vivo* preexisting inflamed niche which can mimic the inflamed conditions found in patients suffering from long-term and recurrent AOM (4) and enables investigation of NTHi infection. Similar to humans, the characteristics (viscosity and opacity) of middle ear fluid from the *Junbo* mouse with OM vary, and this variability can directly affect the ability of NTHi to survive (5). In the present study, we specifically analyze the more viscous/opaque middle ear fluids that support NTHi infection (5) in order to understand the immune cell dynamics induced by the presence of the bacteria over time. We identify changes in innate and adaptive immune cells over 7 days of NTHi infection in the middle ear. Further, we characterize the cytokine/chemokine response against NTHi infection and uncover aspects of immune cell dynamics that are induced by NTHi in the highly inflamed middle ear.

## RESULTS

### NTHi infection induces cell death in the middle ear fluid.

**FIG 1:**
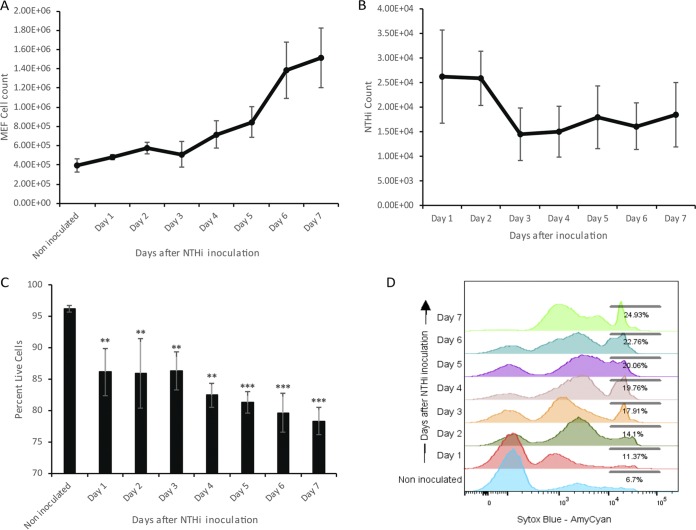
(A) *Junbo* mouse middle ear fluid (MEF) total cell count in noninoculated mice and across 7 days of NTHi infection. (B) NTHi count across 7 days of infection in the MEF of the *Junbo* mouse. (C) Percentage of live cells in noninoculated and NTHi-infected *Junbo* mouse MEF across 7 days of infection. (D) Representative flow cytometry plot, showing an increase in the Sytox blue fluorescent intensity (detected in AmyCyan channel) of MEF cells in noninoculated mice and across 7 days of NTHi infection. The error bars indicate standard errors of mean (*n* = 14; **, *P* < 0.01; ***, *P* < 0.001).

The *Junbo* mouse middle ear has a high cellular content, and analysis of this fluid at 7 days postinoculation with NTHi infection showed a significant increase in cell numbers. In order to understand the cellular dynamics over the time course of middle ear infection with NTHi, we investigated the cellular changes across this 7-day period. A gradual increase in the middle ear fluid cell number was observed following intranasal inoculation and infection of the ear with NTHi (Fig. 1A). The cell number increased from 4 × 10^5^ to 5 × 10^5^ per μl of middle ear fluid after day 3 of NTHi infection. From day 3 to day 7 of infection, a more rapid increase in cell number was observed, from 5 × 10^5^ on day 3 to 1.3 × 10^6^ per μl of middle ear fluid on day 7 post-NTHi inoculation. Correspondingly, the observed average NTHi count was steady at around 2.5 × 10^4^ CFU at days 1 and 2 then dropped at day 3 to 1.5 × 10^4^ CFU per μl of middle ear fluid; this then remained relatively constant in the later days of infection up to day 7 postinoculation (Fig. 1B).

*Junbo* mouse middle ear fluid varies in its nature and cellular content, and this affects NTHi infection in the middle ear. At day 7 postinoculation, NTHi favors the middle ear fluid with a high necrotic cell content. In the present study, we analyzed the effect of NTHi infection on the live/necrotic cell dynamics in the middle ear fluid. Sustained NTHi infection resulted in a gradual increase in the proportion of dead/necrotic cells (Fig. 1C and D). The percentage of live cells significantly decreased from 90 to 85% at day 1 after NTHi inoculation. Later in NTHi infection, the live cell percentage was significantly reduced to 83% on day 4 and 79% on day 7 postinoculation. Along with the increase in the dead cell percentage after NTHi infection, we observed an increase in the intermediate Sytox blue-positive cell population (Fig. 1D). Analysis of this population (see Fig. S1 in the supplemental material) revealed that on average 90% of these cells were neutrophils; this population might be present due to NETosis (neutrophil extracellular traps) induced after NTHi infection. Overall, these observations indicate that sustained NTHi infection increases cell necrosis in the *Junbo* mouse middle ear fluid, and this contributes to a significant amount of free DNA being available in the fluid that could play a critical role in NTHi biofilm formation and survival (7).

### Cellular immune response against NTHi in the *Junbo* mouse middle ear fluid is dominated by neutrophils.

Neutrophils were the major cells responding to NTHi infection in the middle ear of the *Junbo* mouse. At day 1 postinoculation, the percentage of neutrophils in the middle ear fluid increased from 60 to 90% of total live cells (Table 1). At later days of infection, the percentage remained steady between 76 to 86%; the decrease from 90% on day 1 might be due to increased cell necrosis in the middle ear fluid after persistent infection with NTHi. However, when the corresponding percentages were factored in with the total live cell numbers, in the later days (6 and 7) of infection a 10-fold increase in the neutrophil numbers was observed; neutrophils increased from 2.37 × 10^5^ cells per μl at day 1 to 1.17 × 10^6^ to 1.30 × 10^6^ cells per μl at days 6 and 7 of infection. Other immune cell types that were found to be affected by NTHi infection included CD11b^+^ DCs; the percentage decreased gradually from 2.6% at day 1 to 0.2% at day 7. Monocytes and NK cells did not present in such high numbers in the middle ear fluid (0.16 and 0.2% of total live cells in noninoculated mice, respectively), but as a proportion of immune cells they were both significantly reduced as NTHi infection persisted. Similarly, macrophages also comprised only a low percentage of total live cells (0.13%) in the middle ear fluid of the noninoculated mouse, and this proportion significantly decreased over 5 days of NTHi infection but recovered back to starting levels by day 7 of NTHi infection.

**TABLE 1:** Innate immune cells in middle ear fluid across 7 days of NTHi infection in the middle ear of *Junbo* mouse

MEF immune cells	% total live cells (mean ± SEM)^*a*^							
	NI	Day 1	Day 2	Day 3	Day 4	Day 5	Day 6	Day 7
Granulocyte/neutrophils	59.820 ± 9.7560	**90.075 ± 1.4529**	**79.166 ± 4.0481**	**76.255 ± 10.71**	**81.225 ± 8.3141**	**85.301 ± 6.5253**	**84.310 ± 3.8557**	**85.980 ± 3.9839**
Eosinophils	0.000702 ± 0.000202	0.00232 ± 0.00109	0.00294 ± 0.00034	0.0032 ± 0.001	0.0032 ± 0.0006	0.0011 ± 0.0013	0.0007 ± 0	0.0005 ± 0.0009
Macrophages	0.135 ± 0.028291	***0.046 ± 0.0144***	***0.052 ± 0.0199***	***0.042 ± 0.017***	***0.0004 ± 0.000019***	***0.007 ± 0.0063***	***0.018 ± 0.0095***	0.118 ± 0.0583
Monocytes	0.165 ± 0.016201	***0.117 ± 0.0115***	***0.048 ± 0.0276***	***0.071 ± 0.036***	***0.002 ± 0.0007***	***0.003 ± 0.0011***	***0.022 ± 0.0129***	***0.021 ± 0.0123***
DC_CD8type cells	0.082 ± 0.029694	***0.029 ± 0.0071***	***0.020 ± 0.0081***	***0.038 ± 0.018***	***0.019 ± 0.0091***	***0.002 ± 0.0008***	***0.002 ± 0.0009***	***0.005 ± 0.0031***
DC_CD11btype cells	2.626 ± 0.771444	2.632 ± 0.35764	2.610 ± 0.63955	***1.542 ± 0.393***	***0.721 ± 0.1023***	***0.421 ± 0.1478***	***0.337 ± 0.1114***	***0.237 ± 0.0783***
NK cells	0.225 ± 0.0623	***0.015 ± 0.0019***	***0.008 ± 0.0034***	***0.005 ± 0.001***	***0.005 ± 0.0019***	***0.005 ± 0.0039***	***0.005 ± 0.0021***	***0.004 ± 0.0009***

a Values are the percentages of total live cells. Values in boldface represent changes in the MEF cell percentage after NTHi inoculation; underlined values are significantly increased (*P* < 0.05), whereas values in italics are significantly decreased (*P* < 0.05) compared to noninoculated *Junbo* mice. MEF, middle ear fluid; DCs, dendritic cells; NK, natural killer; NI, noninoculated.

The total leukocyte count in blood from the *Junbo* mouse did not vary after NTHi infection and was steady in the range of 2 × 10^3^ to 3 × 10^3^ cells per μl; however, we observed significant variation in the percentages of several immune cell types. In the blood and spleens sampled from the *Junbo* mouse, the percentages of neutrophils increased after NTHi infection; at day 1 postinoculation the neutrophil percentage in the blood increased from 12.1 to 21.6% (Table 2), whereas in the spleen it increased from 2.5 to 4.9% of total live cells (Table 3). The blood neutrophil percentage increased to 27.4% at day 4 and gradually stabilized back to 13.2% at day 7 of NTHi infection, whereas spleen neutrophil levels increased up to 9.2% at day 5 and decreased somewhat to 7.3% at day 7 of NTHi infection. Macrophages were another immune cell type whose proportions were altered in the blood and spleen after NTHi middle ear infection. The blood macrophage percentage increased from 0.4 to 1.0% of total live cells (Table 2), whereas in the spleen it increased from 2.8 to 7.3% (Table 3) at day 1 of NTHi infection. In the later days of infection, the percentages of macrophages in blood decreased to 0.2% of total live cells. Spleen macrophage numbers were high (6.6%) on day 2 postinoculation and decreased to 2.6% of total live cells over the later days of infection (Table 3).

**TABLE 2:** Innate immune cells in blood over 7 days of NTHi infection in the *Junbo* mouse

Blood immune cells	% total live cells (mean ± SEM)^*a*^							
	NI	Day 1	Day 2	Day 3	Day 4	Day 5	Day 6	Day 7
Granulocyte/neutrophils	12.142 ± 1.09	**21.575 ± 2.38**	**20.035 ± 1.31**	**19.550 ± 5.85**	**27.375 ± 1.10**	**31.725 ± 5.54**	**23.124 ± 4.38**	13.201 ± 1.99
Eosinophils	0.055 ± 0.003	**0.147 ± 0.01**	0.047 ± 0.005	0.066 ± 0.02	**0.196 ± 0.13**	**0.119 ± 0.03**	0.085 ± 0.03	0.046 ± 0.006
Macrophages	0.417 ± 0.025	**1.030 ± 0.17**	0.198 ± 0.08	0.690 ± 0.52	0.177 ± 0.13	0.279 ± 0.15	0.241 ± 0.043	0.210 ± 0.03
Monocytes	5.210 ± 0.73	***4.942 ± 0.44***	***1.352 ± 0.29***	***2.192 ± 0.55***	***2.251 ± 0.01***	***2.302 ± 0.07***	***2.914 ± 0.65***	***3.241 ± 0.31***
DC_CD8type cells	1.962 ± 0.12	**2.322 ± 0.24**	***0.255 ± 0.06***	***0.215 ± 0.09***	***0.245 ± 0.42***	***0.792 ± 0.18***	***0.624 ± 0.06***	***0.640 ± 0.04***
DC_CD11btype cells	0.805 ± 0.12	0.882 ± 0.045	***0.212 ± 0.029***	***0.230 ± 0.11***	***0.286 ± 0.56***	***0.237 ± 0.02***	***0.314 ± 0.07***	***0.490 ± 0.03***
NK cells	0.832 ± 0.09	***0.325 ± 0.03***	***0.114 ± 0.007***	***0.078 ± 0.017***	***0.292 ± 0.06***	***0.091 ± 0.006***	***0.071 ± 0.017***	***0.059 ± 0.009***

a Values are the percentages of total live cells. Values in boldface represent changes in the MEF cell percentage after NTHi inoculation; underlined values are significantly increased (*P* < 0.05), whereas values in italics are significantly decreased (*P* < 0.05) compared to noninoculated *Junbo* mice. NI, noninoculated.

**TABLE 3:** Innate immune cells in spleen over 7 days of NTHi infection in the *Junbo* mouse

Spleen immune cells	% total live cells (mean ± SEM)^*a*^							
	NI	Day 1	Day 2	Day 3	Day 4	Day 5	Day 6	Day 7
Granulocytes/neutrophils	2.547 ± 0.324	**4.877 ± 0.852**	**4.110 ± 0.301**	**7.007 ± 1.489**	**5.987 ± 0.409**	**9.171 ± 2.344**	**8.124 ± 2.703**	**7.325 ± 0.352**
Eosinophils	0.015 ± 0.004	**0.101 ± 0.011**	0.016 ± 0.002	0.041 ± 0.021	0.009 ± 0.001	0.063 ± 0.021	0.045 ± 0.023	0.013 ± 0.001
Macrophages	2.777 ± 0.315	**7.257 ± 0.444**	**6.592 ± 0.486**	2.660 ± 0.441	2.003 ± 0.001	2.131 ± 0.009	1.931 ± 0.783	2.635 ± 0.2714
Monocytes	0.882 ± 0.091	**1.180 ± 0.134**	**1.382 ± 0.109**	**1.662 ± 0.086**	**1.481 ± 0.044**	**2.550 ± 0.503**	**1.952 ± 0.607**	**1.292 ± 0.1602**
DC_CD8type cells	3.122 ± 0.144	***1.907 ± 0.191***	***1.951 ± 0.092***	***1.622 ± 0.268***	***1.782 ± 0.155***	***1.355 ± 0.253***	***1.674 ± 0.331***	***1.812 ± 0.1194***
DC_CD11btype cells	2.512 ± 0.122	***0.577 ± 0.038***	***0.501 ± 0.018***	***0.937 ± 0.104***	***1.342 ± 0.209***	***1.182 ± 0.004***	***1.865 ± 0.196***	***2.072 ± 0.0608***
NK cells	0.395 ± 0.025	0.425 ± 0.041	0.350 ± 0.007	0.272 ± 0.0215	0.221 ± 0.034	0.297 ± 0.107	0.301 ± 0.007	0.343 ± 0.02

a Values are the percentages of total live cells. Values in boldface represent changes in the MEF cell percentage after NTHi inoculation; underlined values are significantly increased (*P* < 0.05), whereas values in italics are significantly decreased (*P* < 0.05) compared to noninoculated *Junbo* mice. NI, noninoculated.

The percentages of monocytes in blood remained constant at around 5% of total live cells on day 1 after infection but decreased to between 1.4 and 3.2% in the later days of infection (Table 2). In the spleen, the percentages of monocytes gradually increased from 0.9% to reach a maximum of 2.6% of total live cells at day 5 postinoculation with NTHi (Table 3). The percentages of CD11b^+^ DCs (DC_CD8type) in blood increased slightly from 2.0 to 2.3% of total live cells at day 1 postinoculation with NTHi but significantly decreased to between 0.2 and 0.8% in the later days of infection. CD11b^+^ DCs in blood remained constant at 0.8% of total live cells at day 1 of NTHi infection but also reduced to between 0.2 and 0.5% up to day 7 postinfection. In spleen, a loss of dendritic cells was observed after NTHi infection; CD11b^+^ dendritic cells (DC_CD8type) decreased from 3.1 to 1.4% of total live cells at day 5 postinoculation with NTHi, whereas CD11b^+^ dendritic cells decreased from 2.5% to 0.5 to 0.6% at days 1 and 2 of infection. The level of CD11b^+^ dendritic cells was restored to just over 2% at day 7 of infection. The NK cell percentage stayed constant at around 0.2 to 0.4% in spleen but gradually reduced from 0.8 to 0.06% of total live cells in blood at 7 days postinoculation with NTHi.

### NTHi infection increases the numbers of T-reg cells in *Junbo* mouse middle ear fluid.

**FIG 2:**
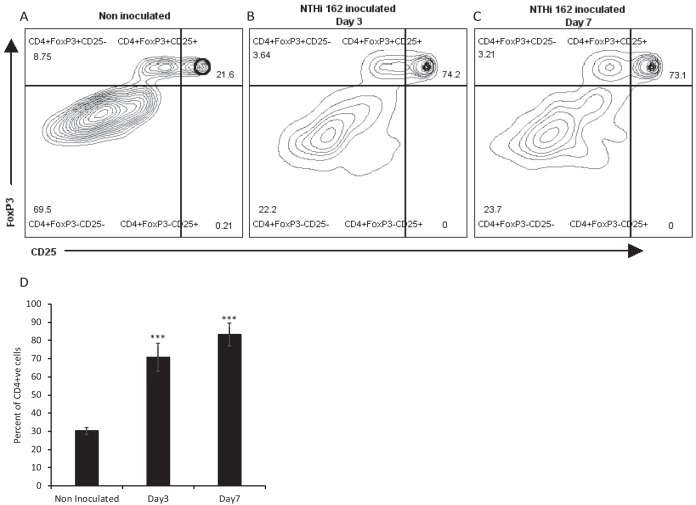
NTHi infection increases T-reg cell population in *Junbo* mouse MEF. Representative flow cytometry plots obtained after analysis of MEF collected from noninoculated (A) and day 3 (B)- and day 7 (C)-NTHi-infected *Junbo* mice are shown. (D) The average percentages of CD4^+^ CD25^+^ FoxP3^+^ T-reg cells in noninoculated and in day 3 and day 7 NTHi-infected *Junbo* mice. The error bars indicate standard errors of mean (*n* = 8; ***, *P* < 0.001).

Along with innate immune cells, we observed dynamic changes in adaptive immune cells in the *Junbo* mouse middle ear fluid following NTHi infection (Table 4). The T-helper (Th) cells (CD4^+^) were the most abundant lymphocytes (1.8% of total live cells) present in the noninfected middle ear fluid and were mostly represented by effector Th (42%) cells (CD44^hi^). After 24 h of NTHi infection, the percentage of Th cells reduced to 0.39% of total live cells and was dominated by effector T-reg (CD44^+^ CD25^+^) cells (74.5%). The percentage of Th cells gradually increased, reaching 6.1% of total live cells at day 5 of NTHi infection; of these, nearly 60% were effector T-reg cells at all time points. To confirm that the CD25^+^ cells observed in the middle ear fluid were in fact T-reg cells, middle ear fluid from *Junbo* mice at day 3 and day 7 postinoculation with NTHi was analyzed for CD4^+^ CD25^+^ FoxP3^+^ cells (Fig. 2). The percentages of CD4^+^ CD25^+^ FoxP3^+^ T-reg cells increased from 21% in noninoculated middle ear fluid to 70% in day 3 and day 7 NTHi-infected middle ear fluid, confirming the induction of T-reg cells by sustained NTHi infection.

**TABLE 4:** Adaptive immune cells in middle ear fluid over 7 days of NTHi infection in the *Junbo* mouse

MEF immune cells	% total live cells (mean ± SEM)^*a*^							
	NI	Day 1	Day 2	Day 3	Day 4	Day 5	Day 6	Day 7
T-helper cells (Th cells)	1.817 ± 0.37085	***0.387 ± 0.0825***	***1.081 ± 0.22339***	1.611 ± 0.53477	**4.214 ± 0.6745**	**6.130 ± 1.62434**	**2.124 ± 1.32141**	0.183 ± 0.03045
Effector T-reg % Th cells	24.485 ± 3.17890	**74.175 ± 12.189**	**58.833 ± 7.55428**	**55.125 ± 6.42097**	**49.214 ± 3.00121**	**51.037 ± 2.79258**	**56.142 ± 1.95247**	**60.628 ± 3.75682**
T-reg % Th cells	4.018 ± 1.10075	***0.887 ± 0.7604***	***2.821 ± 1.28243***	***2.412 ± 1.1735***	***2.123 ± 0.37412***	***2.650 ± 0.88759***	***1.998 ± 1.00063***	***1.645 ± 0.73669***
Effector T-helper % Th cells	68.426 ± 7.44276	***19.601 ± 5.0606***	***32.470 ± 4.50245***	***30.846 ± 5.03286***	***38.741 ± 1.93325***	***40.975 ± 4.52437***	***29.914 ± 3.01214***	***28.357 ± 2.64601***
T-cytotoxic cells (Tcyt cells)	0.741 ± 0.33244	***0.075 ± 0.0325***	***0.103 ± 0.03288***	0.502 ± 0.10762	**0.914 ± 0.21234**	**1.002 ± 0.32923**	**0.841 ± 0.2314**	**1.074 ± 0.02816**
Effector T-cytotoxic % Tcyt cells	66.785 ± 12.5712	**77.450 ± 4.3952**	**79.550 ± 7.56658**	**90.901 ± 5.64623**	**84.994 ± 8.03124**	**86.682 ± 13.2998**	**90.124 ± 4.32145**	**88.585 ± 2.09187**
Naive T-cytotoxic % Tcyt cells	11.160 ± 3.79569	***1.092 ± 0.63352***	***3.881 ± 1.39259***	***2.332 ± 1.14147***	***3.001 ± 0.67412***	***2.203 ± 0.44324***	***3.124 ± 1.00021***	***2.395 ± 1.48838***
Resting T-cytotoxic % Tcyt cells	7.532 ± 2.04648	**16.082 ± 5.1955**	8.955 ± 1.96374	***2.322 ± 1.76877***	***1.947 ± 0.21031***	***0.210 ± 0.20425***	***2.214 ± 1.19474***	***3.788 ± 2.40084***
B2 cells, mature	0.068 ± 0.036409	0.067 ± 0.00699	**0.135 ± 0.062073**	0.057 ± 0.021299	***0.009 ± 0.009145***	***0.014 ± 0.006183***	***0.021 ± 0.014801***	0.088 ± 0.048204
B2 cells, immature	1.156 ± 0.106892	***0.335 ± 0.044197***	1.350 ± 0.452268	***0.683 ± 0.155287***	***0.681 ± 0.093325***	***0.592 ± 0.181023***	***0.295 ± 0.092421***	***0.677 ± 0.248671***
B1 cells	0.808 ± 0.169629	***0.210 ± 0.020331***	1.190 ± 0.483556	0.632 ± 0.190167	0.516 ± 0.210314	0.416 ± 0.111659	***0.222 ± 0.053909***	0.677 ± 0.296533
NK-T cells	0.531 ± 0.22575	***0.016 ± 0.0034***	0.696 ± 0.11244	0.526 ± 0.16758	0.547 ± 0.2141	0.478 ± 0.23145	0.697 ± 0.14745	0.837 ± 0.16545
Effector NK-T cells % NK-T cells	89.014 ± 3.91367	***32.925 ± 5.7041***	**95.183 ± 1.22431**	**96.475 ± 2.18360**	**94.121 ± 4.10231**	**93.462 ± 3.83416**	**96.147 ± 1.01475**	**97.814 ± 0.37380**
Resting NK-T cells % NK-T cells	9.735 ± 4.01887	**65.004 ± 6.9987**	***3.718 ± 1.02736***	***2.851 ± 1.74466***	***3.451 ± 0.91021***	***3.890 ± 2.23951***	***4.112 ± 1.64571***	***1.208 ± 0.25631***

a Values in nonshaded boxes are the percentages of total live cells; the values below in shaded boxes represent the percentages of the subtypes for the respective immune cell type. Values in boldface represent changes in the MEF cell percentage after NTHi inoculation; underlined values are significantly increased (*P* < 0.05), whereas values in italics are significantly decreased (*P* < 0.05) compared to noninoculated *Junbo* mice. NI, noninoculated.

In T-cytotoxic (CD8^+^) (Tcyt) cells, an initial decline was observed in the percentage of the cells from 0.7 to 0.07% of total live cells at day 1 of NTHi infection, which gradually increased to around 1% at days 5, 6, and 7 of NTHi infection (Table 4). Nearly 67% of Tcyt cells in noninoculated middle ear fluid were effector Tcyt (CD44^+^ CD62L^−^) cells, and the percentages of these gradually increased up to 90% in the later days of NTHi infection. In the case of B cells, immature B2 cells dominated (1.16% of total live cells) the middle ear fluid; this decreased to 0.33% after day 1 of NTHi infection. A significant increase to 1.35% in the proportion of these cells was observed at day 2 that decreased to 0.3 to 0.7% in the later days of infection. The percentage of B1 cells in the middle ear fluid was reduced from 0.8 to 0.2% of total live cells at day 1 postinoculation with NTHi; this increased to 1.2% at day 2 and gradually decreased to 0.3 to 0.7% in the later days of NTHi infection. The proportion of natural killer T (NK-T) cells decreased from 0.5 to 0.01% of the total live cells at day 1 postinoculation with NTHi; at day 1 these were mostly dominated by naive NK-T (CD62L^+^) cells (65%). The percentage of NK-T cells present during the later days of infection remained between 0.5 and 0.8% of total live cells and was mostly dominated by effector (CD62L^–^) cells (93 to 98%).

The percentage of Th cells in the spleen gradually increased from 16.2 to 20.6% of total live cells at day 5 after NTHi infection (Table 5). In spleens from noninoculated mice, only 12% of the Th cells were T-reg cells (CD25^+^ CD44^+^), whereas after NTHi inoculation the percentage of these cells increased to between 27 and 44%. The Tcyt cell percentage increased from 7.6 to 11.8% at day 4 of NTHi infection, and the majority of these cells were naive Tcyt (CD44^−^ CD62L^+^) cells. The percentage of NK-T cells increased from 0.3 to 1.8% after 1 day of NTHi infection and decreased to between 0.8% and 0.9% in the later days of infection. Approximately 70% of these NK-T cells were naive (CD62L^+^) cells. The proportion of splenic B cells did not vary significantly after NTHi infection of the middle ear.

**TABLE 5:** Adaptive immune cells in spleen over 7 days of NTHi infection in the *Junbo* mouse

Spleen immune cells	% total live cells (mean ± SEM)^*a*^							
	NI	Day 1	Day 2	Day 3	Day 4	Day 5	Day 6	Day 7
Th cells	16.225 ± 0.286865	**18.051 ± 0.53619**	**18.025 ± 0.85865**	**17.975 ± 0.8984**	**20.550 ± 0.2958**	**20.625 ± 0.90404**	**20.124 ± 0.85865**	**19.733 ± 0.46188**
Effector T-reg % Th cells	12.050 ± 0.298608	**44.225 ± 0.43277**	**37.501 ± 2.26163**	**31.310 ± 1.2172**	**36.595 ± 0.2136**	**27.397 ± 0.63488**	**29.124 ± 2.26163**	**31.866** **± 1.32790**
T-reg % Th cells	0.660 ± 0.056716	**15.775 ± 1.24392**	**9.742 ± 1.24718**	**7.962 ± 1.2835**	**6.627 ± 0.0884**	**7.872 ± 0.14343**	**8.014 ± 1.24718**	**9.766 ± 1.10943**
Effector T-helper % Th cells	82.951 ± 1.034811	***30.225 ± 0.54064***	***39.750 ± 2.34822***	***47.275 ± 1.1301***	***57.651 ± 0.4991***	***56.775 ± 1.53263***	***52.203 ± 2.34822***	***42.401 ± 1.52561***
T-cytotoxic cells (Tcyt cells)	7.642 ± 0.2675	7.497 ± 0.37761	**8.272 ± 0.51220**	**8.422 ± 0.2254**	**11.850 ± 0.3403**	**9.751 ± 0.64593**	**9.812 ± 0.5122**	**8.856 ± 0.20774**
Effector T-cytotoxic % Tcyt cells	6.067 ± 1.071342	7.880 ± 0.72570	6.721 ± 0.58843	***4.201 ± 0.50264***	***2.865 ± 0.2456***	***2.815 ± 0.31319***	***4.215 ± 0.58843***	7.383 ± 0.54805
Naive T-cytotoxic % Tcyt cells	72.925 ± 0.623665	***60.425 ± 1.41384***	***61.601 ± 0.98910***	***67.775 ± 1.16574***	***66.925 ± 0.31983***	72.875 ± 0.91957	69.212 ± 0.98911	61.066 ± 0.88928
Resting T-cytotoxic % Tcyt cells	18.175 ± 0.705189	**24.525 ± 0.62232**	**20.650 ± 0.46636**	18.301 ± 1.20485	18.112 ± 0.24438	18.025 ± 0.31983	17.124 ± 0.46637	16.207 ± 0.21794
B2 cells, mature	1.170 ± 0.036286	1.705 ± 0.09925	1.657 ± 0.06908	1.265 ± 0.35741	1.150 ± 0.00912	1.617 ± 0.07767	1.314 ± 0.38628	1.711 ± 0.08808
B2 cells, immature	1.837 ± 0.053288	1.112 ± 0.02982	1.227 ± 0.10633	1.605 ± 0.59588	1.545 ± 0.48272	1.412 ± 0.06663	1.132 ± 0.01707	**2.340 ± 0.13714**
B1 cells	13.72 ± 2.54981	10.22 ± 4.028	12.97 ± 1.6169	9.87 ± 3.8673	9.47 ± 4.89849	12.21 ± 1.732	13.14 ± 3.1522	**16.52 ± 2.09776**
NK-T cells	0.295 ± 0.022546	**1.772 ± 0.0825**	**0.985 ± 0.04031**	**0.935 ± 0.03617**	**0.812 ± 0.02561**	**0.782 ± 0.03705**	**0.820 ± 0.04031**	**0.896 ± 0.02929**
Effector NK-T cells % NK-T cells	39.004 ± 3.371202	***23.875 ± 4.50954***	***29.301 ± 1.96426***	33.706 ± 5.32071	37.850 ± 1.72747	45.350 ± 14.7473	30.142 ± 1.96426	28.333 ± 1.76941
Resting NK-T cells % NK-T cells	53.351 ± 3.064175	**73.725 ± 4.61868**	**67.950 ± 1.64848**	**63.550 ± 5.98894**	**77.575 ± 1.00114**	**63.675 ± 5.2682**	**59.213 ± 1.64848**	**69.033 ± 2.56141**

a Values in nonshaded boxes are the percentages of total live cells; the values below in shaded boxes represent the percentages of the subtypes for the respective immune cell type. Values in boldface represent change in MEF cell percentage after NTHi inoculation; underlined values are significantly increased (*P* < 0.05), whereas values in italics are significantly decreased (*P* < 0.05) compared to noninoculated *Junbo* mice. NI, noninoculated.

At day 1 after NTHi inoculation, the percentages of Th cells, Tcyt cells, and B lymphocytes in blood did not show substantial variation from the noninfected mouse (Table 6). An increase in the percentage of NK-T cells from 0.06 to 1.54% of the total live cells was observed at day 1 postinoculation with NTHi. At the later times of infection, the percentage of NK-T cells ranged between 0.4 and 0.8%, and nearly 65% of these cells were effector cells (CD62L^–^).

**TABLE 6:** Adaptive immune cells in blood over 7 days of NTHi infection in the *Junbo* mouse

Blood immune cells	% total live cells (mean ± SEM)^*a*^							
	NI	Day 1	Day 2	Day 3	Day 4	Day 5	Day 6	Day 7
Th cells	10.482 ± 2.13	10.597 ± 1.24	11.005 ± 1.18	7.401 ± 2.84	10.220 ± 0.56	10.302 ± 0.36	8.141 ± 0.84	7.587 ± 1.13
Effector T-reg % Th cells	7.515 ± 0.78	10.970 ± 0.67	8.632 ± 1.21	7.121 ± 0.59	***3.617 ± 0.71***	***3.160 ± 0.23***	***4.131 ± 0.59***	6.532 ± 0.61
T-reg % Th cells	0.855 ± 0.081	0.774 ± 0.05	0.702 ± 0.08	0.872 ± 0.11	**1.792 ± 0.18**	**1.597 ± 0.20**	0.912 ± 0.11	0.727 ± 0.09
Effector Th % Th cells	66.201 ± 2.20	57.357 ± 0.89	61.807 ± 2.21	59.575 ± 1.26	61.351 ± 1.13	62.257 ± 0.62	60.213 ± 1.26	59.275 ± 1.06
T-cytotoxic cells (Tcyt cells)	4.762 ± 0.77	4.840 ± 0.60	4.822 ± 0.60	4.961 ± 0.37	4.133 ± 0.29	4.114 ± 0.27	***3.125 ± 0.37***	***2.882 ± 0.37***
Effector T-cytotoxic % Tcyt cells	17.437 ± 7.5	18.325 ± 1.91	18.625 ± 1.45	***11.547 ± 1.05***	***8.967 ± 2.71***	***13.095 ± 0.45***	***12.141 ± 1.05***	***13.662 ± 2.38***
Naive T-cytotoxic % Tcyt cells	49.425 ± 3.98	***38.301 ± 1.50***	***38.875 ± 2.00***	46.825 ± 2.06	**66.875 ± 2.77**	**77.725 ± 0.59**	**57.41 ± 2.06**	47.625 ± 2.55
Resting T-cytotoxic % Tcyt cells	19.575 ± 2.31	23.451 ± 0.93	22.625 ± 0.59	21.975 ± 1.33	22.851 ± 1.61	17.701 ± 0.89	23.141 ± 1.33	25.275 ± 1.10
B2 cells, mature	0.332 ± 0.018	**0.585 ± 0.055**	***0.129 ± 0.036***	***0.137 ± 0.05***	***0.164 ± 0.004***	0.301 ± 0.04	0.213 ± 0.015	0.291 ± 0.02
B2 cells, immature	0.592 ± 0.077	0.607 ± 0.08	0.637 ± 0.02	0.682 ± 0.03	0.917 ± 0.32	0.581 ± 0.09	0.647 ± 0.12	0.172 ± 0.3
B1 cells	0.102 ± 0.025	***0.097 ± 0.019***	***0.015 ± 0.005***	***0.011 ± 0.003***	***0.095 ± 0.0026***	***0.095 ± 0.02***	***0.078 ± 0.02***	***0.025 ± 0.005***
NK-T cells	0.065 ± 0.007	**1.540 ± 0.08**	**0.832 ± 0.06**	**0.762 ± 0.17**	**0.522 ± 0.07**	**0.457 ± 0.09**	**0.617 ± 0.17**	**0.731 ± 0.11**
Effector NK-T cells % NK-T cells	74.450 ± 3.01	***61.375 ± 5.47***	***54.325 ± 4.13***	***68.175 ± 2.71***	74.201 ± 2.62	75.525 ± 4.24	73.211 ± 2.71	70.325 ± 3.87
Resting NK-T cells % NK-T cells	24.611 ± 3.02	**37.251 ± 5.35**	**44.609 ± 4.26**	30.601 ± 2.59	23.825 ± 2.36	23.418 ± 4.79	26.420 ± 2.59	29.111 ± 3.77

a Values in nonshaded boxes are the percentages of total live cells; the values below in shaded boxes represent the percentages of the subtypes for the respective immune cell type. Values in boldface represent changes in MEF cell percentages after NTHi inoculation; underlined values are significantly increased (*P* < 0.05), whereas values in italics are significantly decreased (*P* < 0.05) compared to noninoculated *Junbo* mice. NI, noninoculated.

### Cytokine/chemokine dynamics against NTHi infection in the middle ear.

**FIG 3:**
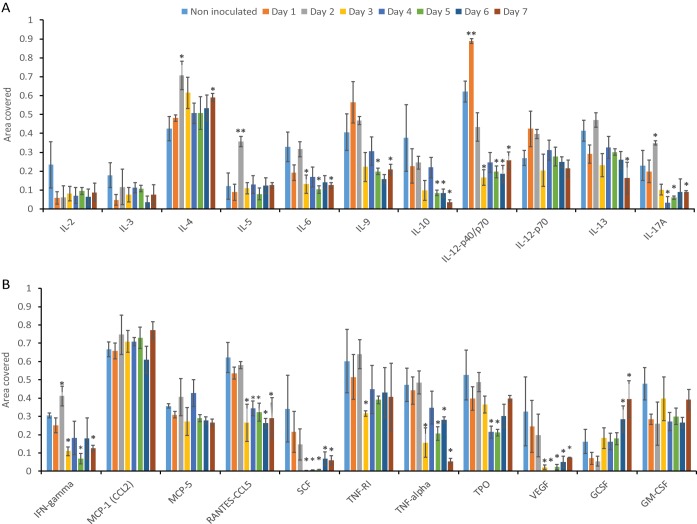
Cytokine and chemokine levels in the *Junbo* mouse MEF after NTHi infection. (A and B) The area coverage represents the cytokine/chemokine levels obtained after analyses of the noninoculated and NTHi-infected *Junbo* mouse MEF across 7 days of infection. The area coverage was calculated using ImageLab (Bio-Rad) analysis on the RayBioRc C-Series mouse cytokine antibody array C1. A value of 1 represents the area coverage of the positive control on the immunoblot. The error bars indicate standard errors of mean (*n* = 4; *, *P* < 0.05; **, *P* < 0.01).

**FIG 4:**
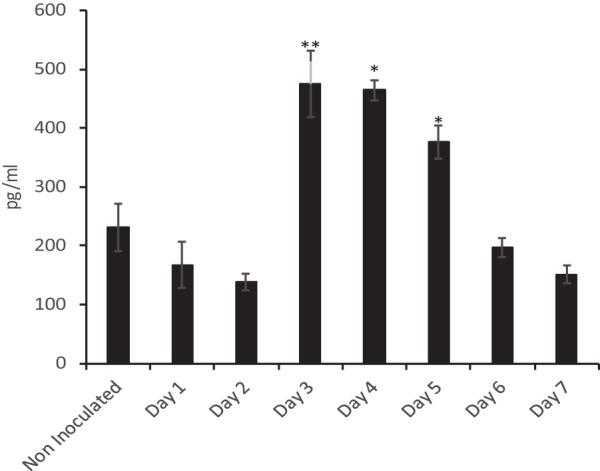
Sustained NTHi infection increases the TGF-β levels in the *Junbo* mouse MEF. TGF-β levels in the MEF were measured in noninoculated and NTHi-infected *Junbo* mice across 7 days of infection, using mouse TGF-β 1 DuoSet ELISA (R&D Systems). The concentrations (in pg/ml) were obtained from a standard graph of concentration against absorbance at 450 nm. The error bars indicate standard errors of mean (*n* = 6; *, *P* < 0.05; **, *P* < 0.01).

NTHi infection in the middle ears of *Junbo* mice leads to immune cell infiltration in the middle ear fluid. To understand the influence of the autocrine and paracrine signals post-NTHi infection, we analyzed the specific cytokines/chemokines that modulate the functional response in these cells. After NTHi infection, we monitored the changes in cytokine/chemokine levels in the infected middle ear fluid over 7 days. The noninfected middle ear fluid of *Junbo* mice had high levels of IL-4, IL-9, IL-12p40/p70, CCL2, TNF-RI, TNF-α, thrombopoietin (TPO), RANTES-CCL5, granulocyte-macrophage colony-stimulating factor (GM-CSF) (Fig. 3), and transforming growth factor β (TGF-β) (Fig. 4). The levels of CCL2 were constantly high in the middle ear fluid before and after NTHi infection. At day 1 of NTHi infection, a significant increase in IL-12p40/p70 was observed in the middle ear fluid, whereas the IL-12p70 levels were slightly increased. The IL-12p70 cytokine is composed of two heterodimers: p40 and p35. In the dot blot analysis, the IL-12p40/p70 level includes both p40 and the p40+p35 complex, whereas IL-12p70 analysis measures only the level of the active heterodimer (i.e., p40+p35) complex. The significant increase in IL-12p40/p70 compared to IL12p70 indicates an increase in IL-12p40 levels at day 1 of NTHi infection. No other cytokine/chemokine significantly changed at day 1 of NTHi infection. At day 2 of NTHi infection, the level of the major Th2-dependent cytokine, IL-4, was significantly increased and then remained high in the later days of NTHi infection (Fig. 3). Other cytokines that were slightly elevated at day 2 of infection were Th17-dependent IL-17A, Th1-dependent gamma interferon (IFN-γ), and IL-5. At day 3 of NTHi infection, TGF-β was the major cytokine that was significantly elevated (Fig. 4); this correlated with the observed increase in the T-reg cell population and a dampening of other cytokine/chemokine levels in the middle ear fluid. A substantial decrease in IL-6, IL-9, IL-12p40/p70, IL-13, IL-17, IFN-γ, CCL5, SCF, TNF-α, TPO, and vascular endothelial growth factor (VEGF) was observed across days 4 through 7 of NTHi infection (Fig. 3). Despite the rise in the T-reg cell population, the levels of the major T-reg-dependent cytokine, IL-10, were substantially decreased after day 3 of NTHi infection. TGF-β returned to earlier levels found in noninfected middle ear fluids at days 6 and 7 of NTHi infection. The chemokine levels that increased during the later days (6 and 7) of NTHi infection were only those of G-CSF; this correlates with the increase in granulocyte levels observed in the middle ear fluid. In summary, the major fluctuations in cytokine/chemokine dynamics relevant to the observed changes in immune cells after NTHi infection of the *Junbo* mouse middle ear were for IL-12p40/p70, IL-4, and TGF-β.

## DISCUSSION

In the present study, we investigated the cellular and immune changes induced due to NTHi infection in the inflamed middle ear utilizing a well-established *Junbo* mouse model of OM (16); a single intranasal inoculation with NTHi causes sustained infection in a preexisting inflamed middle ear (17). Previously, we reported that variability in the composition of the *Junbo* mouse middle ear fluid influences NTHi infection rate and the titer attained in this compartment in the mouse host (5). Adopting a similar infection strategy, the changes in the middle ear fluid with respect to NTHi infection, live/dead cells, immune cells, and cytokine/chemokine composition were analyzed over 7 days of NTHi infection. The noninoculated *Junbo* mouse middle ear fluid was rich in neutrophils and necrotic cells, and NTHi infection increased the proportion of neutrophils and induced more cell necrosis. NTHi is known to induce neutrophil extracellular trap formation (9) and is able to form a biofilm in the extracellular DNA and other material released as a result of this process (18); DNA and other released material may also act as nutrients to facilitate NTHi growth. The cell necrosis was observed across 7 days of sustained NTHi infection (Fig. 1). A similar significant increase in neutrophil numbers was observed in blood; this may be the source of neutrophils that migrate to the middle ear in response to the presence of NTHi (19). The macrophage was another cell type whose numbers were elevated in both blood and spleen following NTHi inoculation. A major increase in the proportion of macrophages in the spleen was observed on day 5 of NTHi infection, indicative of systemic lymphatic drainage for the purification of the blood in the major secondary lymphoid organ (20). Another innate immune cell, the monocyte, can significantly vary in number according to *Junbo* mouse fluid characteristics and shows important correlation with NTHi infection (5); the percentage of this cell type was significantly reduced in the middle ear fluid and blood upon persistent NTHi infection across 7 days.

The major cytokine that responded on day 1 of NTHi infection was IL-12p40. Antigen-presenting cells, such as DCs, are the major source of IL-12p40 that plays a significant role in the Th cell response (21). Nearly 2.6% of live cells in noninoculated *Junbo* middle ear fluid were DCs, and the presence of NTHi did not change this proportion over 2 days following inoculation. After day 1 of infection, dendritic cells are a likely source of IL-12p40 monomer and IL-12p70 heterodimer secretion (22) in the middle ear. IL-12p70 heterodimer induces a Th1-dependent response that promotes IFN-γ production (23). The IL-12p40 monomer and homodimer have an inhibitory effect on Th1 development and a stimulatory effect on Th2 development (24). The noninoculated *Junbo* mouse middle ear had high levels of IL-4, a cytokine that is mainly secreted by Th2 cells (25). At day 2 of NTHi infection, the IL-4 levels significantly increased above the base value for middle ear fluid in the noninoculated *Junbo* mouse. In contrast, the Th1-dependent IFN-γ (25) and Th17-dependent IL-17 levels (26) were significantly lower than those for IL-4. The polyfunctional IFN-γ- and IL-17-producing CD4^+^ T cells in the middle ear of transcutaneous immunized chinchilla, an established model of AOM, clear the NTHi infection (27). These observations indicate that the effector Th cellular response induced after day 2 of NTHi infection in the *Junbo* mouse was Th2 dominant and mainly modulated by IL-12p40 and IL-4 secretion.

Another Th2-dependent cytokine that showed a significant increase in levels at day 2 postinoculation with NTHi was IL-5 (28); this returned to baseline levels at day 3. IL-5 is the major eosinophil and mast cell chemotactic cytokine and plays a critical role in allergy and OM (28–30). The eosinophils did not comprise a significant proportion of the cell population but were classified with our current flow cytometry panel; the presence of mast cells cannot be ruled out. Mast cells have been shown to be present in significant numbers in bacterium-induced OM (30). The role of mast cells in inflammation, infection, and the allergic response (31) indicates that these cells might play a significant role in the pathogenesis of AOM. The noninoculated *Junbo* mouse middle ear fluid contained the cytokine stem cell factor (SCF) that plays a major role in modulation of mast cell function (31, 32); NTHi infection reduced the level of SCF after 3 days of infection. In the present study, even with our extensive flow cytometry panel, it was not possible to add any additional mast cell markers to the profiles. Further analysis of the mast cell response, specifically at day 2 of NTHi infection in the *Junbo* mouse middle ear, could provide additional insight into their role in the pathogenesis of AOM.

At day 3 of NTHi infection, the adaptive immune cell dynamics changed. Noninoculated *Junbo* mouse middle ear fluid had high levels of TGF-β. NTHi infection lowered the TGF-β levels for 2 days, thus preventing immunoregulatory signaling through this cytokine. At day 3 postinoculation with NTHi, a dramatic increase in TGF-β levels was observed that paralleled the rise in the T-reg cell population; these cells play a significant role in dampening the immune response (33). An increase in the level of T-reg cells was observed at days 4 and 5 of NTHi infection; this corresponded with a decrease in the percentages of innate immune cells, increased cell necrosis, and dampening of cytokine/chemokine levels (33). Interestingly, despite the increase in T-reg cells, the IL-10 levels did not increase; IL-10 is one of the major cytokines produced by the T-reg cells (33). Two distinct populations of T-reg cells exist: Tr1 cells, the major IL-10 secreting T-reg cells, and Th3 cells, the major TGF-β-secreting cells, which also secrete variable levels of IL-4 and IL-10 (34). The presence of high TGF-β levels and low IL-10 levels in *Junbo* mouse middle ear fluids indicates that the T-reg cells in the *Junbo* mouse middle ear were mainly of the Th3 type. In addition, the middle ear fluid of the *Junbo* mouse has consistently high levels of IL-4 during NTHi infection. The initial rise in IL-4 levels might be due to a Th2 response, but the increased levels observed at later times of infection indicate a contribution from Th3 T-reg cells. Hirano et al. (15), in their analysis of T-reg cell response induced by NTHi in a mouse OM model induced by blocking the Eustachian tube, observed increased IL-10 levels. Our lack of an IL-10 response might be due to the preexisting inflamed environment that the Evi1 mutation induces in the *Junbo* mouse middle ear (16), which plays a critical role in immune modulation. The second zinc finger domain of Evi1 is essential for both the activation of activator protein 1 (AP-1) and transactivation of the c-*fos* promoter (35). c-Fos belongs to the Fos protein family and binds to c-Jun to form AP-1, one of the important transcriptional factors of the immune system (36, 37). The inhibition of c-*fos* during Brucella abortus macrophage infection leads to an increase in IL-10 levels and immune suppression (38). The effect of the *Junbo* mouse Evi1 mutation on c-*fos* mediated IL-10 modulation is not clear, but it could be postulated as a major contributor in decreasing IL-10 levels, even with the observed increase in T-reg cell numbers. Evi1 also represses TGF-β signaling via Smad3; the mutation in Evi1 affects the TGFβ/Smad3 pathway, which plays a critical role in increasing TGF-β levels and inducing colonic cancer in the absence of IL-10 (39). TGF-β and IL-10 play important roles in immune modulation and inflammation, but cross talk between these two cytokines is still unclear. The *Junbo* mouse Evi1 mutation might affect the molecular pathway for both of these cytokines and could prove to be a useful tool to dissect the mechanism(s) involved.

NTHi inoculation increased the proportion of CD8^+^ T-cytotoxic (Tcyt) cells in the middle ear of the *Junbo* mouse. The percentage of CD8^+^ T cells was significantly lower than that for CD4^+^ T cells. A similar increase in the CD4^+^ compared to CD8^+^ T cells was observed in other mouse models of infection (15) in which AOM was induced by NTHi. Even with low levels of IL-2, a major cytokine secreted and responsible for activation of T-cytotoxic cells (40), an increase in effector Tcyt (CD8^+^ CD44^+^ CD62L^−^) cells was observed in the *Junbo* mouse middle ear due to NTHi infection. Along with the IL-2-dependent pathway, Tcyt cells activate via the contact-dependent mechanism in the presence of MHC-II^+^ antigen-presenting cells, such as dendritic cells (41). The percentage of dendritic cells in OM-prone children is elevated compared to that in non-OM-prone children; this indicates a role in human OM (42). The percentage of dendritic cells in the middle ear fluid of *Junbo* mice did not vary over the first 2 days of infection with NTHi, but an increase in IL-12p40 levels indicates dendritic cell activation that increases the MHC-II expression (43), which supports the activation of the CD8^+^ T cell to effector function. The effector Tcyt cells also secrete granzymes and perforin molecules that cause the death of infected host cells (44), and the cell analysis showed a gradual increase in cell death after NTHi infection. The granzymes and perforin molecules released from the effector Tcyt cells might be contributing factors that induce the observed cell death following NTHi infection in the *Junbo* mouse middle ear. The analysis of systemic response in the blood and spleen in the early days of NTHi middle ear infection showed an increase in the Tcyt cells, and the majority of these cells were naive. In the present study, we rigorously followed up the NTHi infection rate and bacterial titer in the middle ear of the *Junbo* mice, but infection in any other organ/site of the mice was not analyzed. Thus, although it is the most likely scenario, the systemic response observed in *Junbo* mice to intranasal inoculation with NTHi cannot be confirmed as solely being due to middle ear infection.

At the later days (6 and 7) of NTHi infection, the levels of G-CSF in the *Junbo* mouse middle ear fluid significantly increased compared to those in noninoculated mice. G-CSF plays a critical role in inducing neutrophil chemotaxis and activation (45). The Evi1 mutation might again be one of the significant factors leading to the high G-CSF and high neutrophil levels in the *Junbo* mouse. Evi1 suppresses G-CSF-mediated myeloid differentiation and activation of granulocytes (46). The way in which the Evi1 mutation in the *Junbo* mice affects the G-CSF control of neutrophil function is unclear and is under investigation. Along with G-CSF, the level of another proinflammatory cytokine, CCL2, was also high in the middle ear fluid. Earlier we reported that NTHi infects the high opacity/viscosity *Junbo* middle ear fluids that consistently have the highest CCL2 levels (5). From our current analysis, we can assume that NTHi infection does not change the levels of this chemokine in the inflamed middle ear fluid of *Junbo* mice. Lung infection with Escherichia coli in MCP^−/−^ (CCL2-null) mice showed a defect in neutrophil recruitment and function, indicating the significance of this chemokine in the recruitment of neutrophils (47). The G-CSF treatment of Klebsiella pneumoniae-infected MCP^−/−^ mice promotes neutrophil infiltration in the lungs and rescues these mice from infection (48). Thus, high levels of these two proinflammatory cytokines might be an important factor that contributes to maintaining high neutrophil levels and the inflamed state in the *Junbo* mouse middle ear.

As far as we know, the present study is the first attempt to comprehensively elucidate the cellular and molecular immune changes that occur in response to NTHi infection in the preexisting inflamed middle ear environment. In humans, middle ear inflammation can be induced by viruses ascending through the Eustachian tube and this can pave the way for otopathogens, like NTHi, to infect (49). Most of the cellular profiling carried out with other AOM animal models characterize the naive immune response to the presence of NTHi, whereas we have investigated the immune dynamics in the preinflamed environment that mimics the scenario in the progression and recurrence of otitis media in humans associated with NTHi infection. Even in the highly inflamed environment, the neutrophils are the first cells to respond to NTHi infection in the middle ear. Along with continuous depletion of other innate immune cells, a Th2-dependent immune response developed during the initial days of NTHi infection. In the later days of infection, an increase in immune suppressor T-reg cells was observed with increased levels of TGF-β in the NTHi-infected middle ear fluid of *Junbo* mice. These observations that, upon NTHi infection, a continuous induction of the inflammatory immune response is followed by a tolerogenic immune response would provide a possible explanation for long-term NTHi infection and avoidance of a significant memory-dependent immune response to the middle ear infection. Currently, we are investigating the NTHi components that can modulate these immune responses and finding ways to determine vaccine targets that could promote improved bacterial clearance.

## MATERIALS AND METHODS

### Ethics statement.

The humane care and use of mice in this study were carried out under UK Home Office Project license no. PPL3003280 (Studies of Otitis Media in Mouse Models [MRC Harwell Institute code 30/3280]). The protocols, intranasal inoculation of mice (1078/MLC), preparation of Harwell gaseous anesthetic equipment (1104/MLC), and intraperitoneal injection (1083/MLC) were approved by the MRC Harwell Institute Animal Welfare Ethical Review Body (AWERB).

### Mice and bacterial strains.

The heterozygote *Junbo* mouse (*Jbo/+*; here termed *Junbo*) was congenic on a C3H/HeH background. The mice were specific pathogen free and had normal commensal flora. Nontypeable Haemophilus influenzae (NTHi) strain 162 sr (streptomycin resistant) (17) was grown in brain heart infusion (BHI) media supplemented with Leventhal reagent. Prior to intranasal inoculation, NTHi 162 sr was washed with phosphate-buffered saline (PBS) and then resuspended in PBS–1% gelatin (17).

### *Junbo* mouse infection with NTHi.

The inoculation of the *Junbo* mice was carried out as described previously (17). Briefly, *Junbo* mice were inoculated intranasally under gas anesthesia with 5 μl per nares of NTHi 162 sr cell suspension at a concentration of 10^8^ CFU/ml in PBS–1% gelatin. Middle ear fluid was collected across 7 days postinoculation from mice under terminal anesthesia induced by an intraperitoneal overdose of sodium pentobarbital. After removal of any material on the external surface of the tympanic membrane, a hole was made in the membrane while removing the malleus from the middle ear using a sterile pair of forceps. Middle ear fluid was collected with a pipette and microtip, and the middle ear fluid was graded under a microscope on the basis of its viscosity and opacity during collection. Middle ear fluid volume was measured by collecting up to 0.5-μl aliquots multiple times, and the total volume generally ranged between 0.25 and 1.5 μl. Middle ear fluid was collected into 100 μl of PBS buffer, and a portion of it was serially diluted in PBS and plated on BHI agar supplemented with streptomycin (300 μg/ml). The bacterial titer was calculated from the colony count after overnight growth on the plates. The remainder of the fluid was centrifuged at 800 × *g* for 2 min, and the cell pellet was used for flow cytometry, whereas the supernatant was used for cytokine/chemokine analyses. Bloods were collected by retroorbital bleeding under terminal anesthesia in lithium heparin tubes. The total leukocyte count was obtained using an ADVIA 2120i hematology analyzer. Prior to flow cytometry analysis, 50 μl of blood was resuspended in 1 ml of red blood cell lysis buffer (BioLegend) for 10 min on ice, followed by two washes with 1 ml of PBS. The final pellet was resuspended in fluorescence-activated cell sorting (FACS) buffer (5 mM EDTA and 0.5% fetal calf serum in PBS) for flow cytometry analysis. Spleens were collected in gentleMACS C tubes with 3 ml of RPMI supplemented with 10 μg/ml collagenase II (Serlabo) and 166 μg/ml DNase I (Sigma). The spleen was homogenized in a gentleMACS dissociator, and a single cell suspension was separated from tissue debris by passage through a 70-μm-pore-size cell strainer. The cell pellet was obtained after centrifugation at 800 × *g* for 2 min and resuspended in FACS buffer for flow cytometry analysis.

### Flow cytometry analysis.

Middle ear fluid, blood, and spleen samples were diluted with FACS buffer, and a cell count was obtained. The samples were transferred to a 96-well plate at a concentration of 2 × 10^5^ cells/well. Cells were centrifuged and washed twice with FACS buffer. For analysis of different types of immune cells, the suspension was incubated for 15 min with CD16/CD32 antibody (BD Pharmingen) at a dilution of 1:100. After centrifugation at 800 × *g* for 1 min, the cell pellets were resuspended in 100 μl of either of panel 1 or panel 2 (see Table S1 in the supplemental material) antibody cocktail and incubated for 20 min in the dark. Cells were centrifuged and washed twice with FACS buffer and finally resuspended in 100 μl of Sytox DNA stain (1:10,000). After making up the final volume to 250 μl with FACS buffer, flow cytometry was performed using a BD FACSCanto II system. FlowJo software (Tree Star) was used to analyze the data obtained (Fig. S2 and S3; Table S2).

For T-reg cell subtype confirmation, intracellular analysis was performed. Cells from middle ear fluids were fixed with 200 μl of BD CytoFix solution for 20 min, followed by three washes with FACS buffer. Permeabilization of the cells was carried out in 200 μl of BD Prem/Wash solution for 15 min and then stained with 100 μl of antibody cocktail-panel 3 (Table S1) prepared in BD Prem/Wash solution. Cells were washed three times with FACS buffer and finally resuspended in 250 μl of FACS buffer. The flow cytometry of the stained cell population was carried out on a BD FACSCanto II system and analyzed using FlowJo software.

### Cytokine and chemokine responses.

Cytokine and chemokine levels were measured in middle ear fluids collected from NTHi-infected *Junbo* mice (*n* = 3) by using a mouse cytokine antibody array (mouse cytokine array C1; RayBiotech, Inc.) according to the manufacturer’s protocol. The following cytokines and chemokines were analyzed: G-CSF, GM-CSF, and IL-2, IL-3, IL-4, IL-5, IL-6, IL-9, IL-10, IL-12p40/p70, IL-12p70, IL-13, and IL-17, as well as IFN-γ, monocyte chemoattractant proteins 1 and 5 (MCP-1 and MCP-5), RANTES (T cell expressed and secreted), stem cell factor (SCF), soluble TNF receptor type I (sTNFRI), TNF-α, TPO, and VEGF. The signal intensities were quantified using the Image Lab software (Bio-Rad). Changes in middle ear fluid TGF-β levels were analyzed by Mouse TGF-β 1 DuoSet ELISA (R&D Systems) according to the manufacturer’s instruction. The color change was measured at 450 nm on an Epoch BioTek plate reader, and the TGF-β concentration in middle ear fluid was calculated by using a standard curve.

### Data analysis.

We used the unpaired *t* test assuming equal variance for comparing the mean percentages of dead cells, immune cells, and cytokine/chemokine changes after NTHi infection. A *P* value of <0.05 was considered significant.

## Supplementary Material

Supplemental file 1
