# Supplementary material for: Cellular Immune Response against Nontypeable Haemophilus influenzae Infecting the Preinflamed Middle Ear of the Junbo Mouse
Source: Infect Immun. 2019 Nov 18;87(12):e00689-19. doi: 10.1128/IAI.00689-19 (PMC6867859; doi:10.1128/IAI.00689-19)
Supplement: Supplemental file 1 [file IAI.00689-19-s0001.pdf]

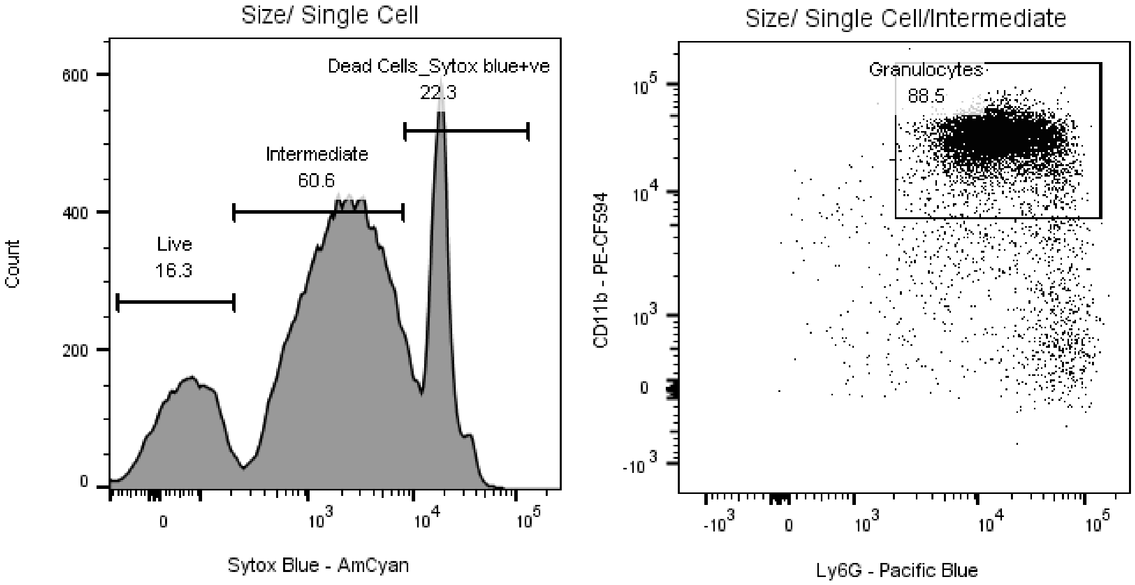

Fig S1: Analysis of Sytox blue histograms for neutrophil content.

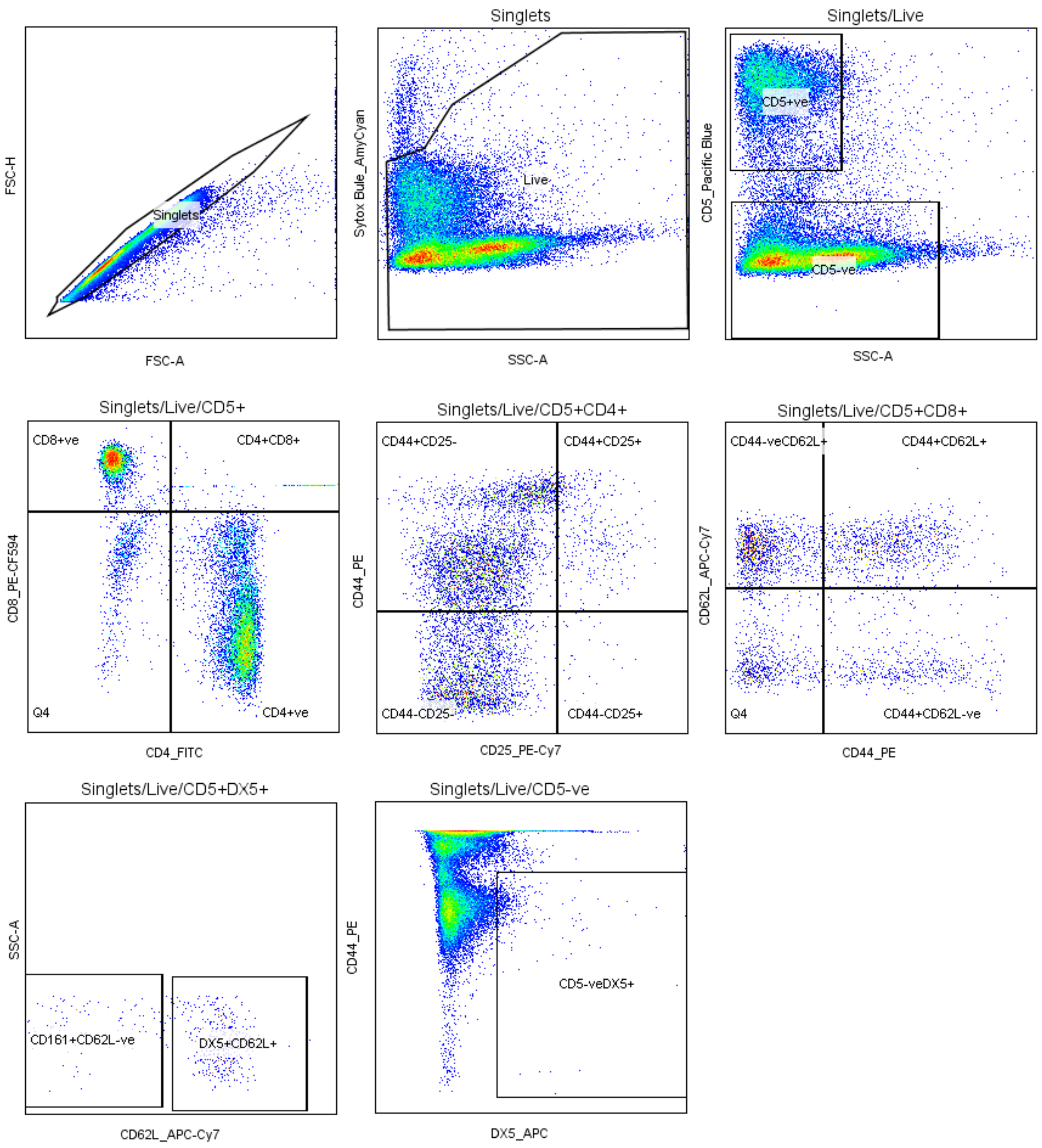

Fig S2: Flow cytometry panel 1 gating strategy.

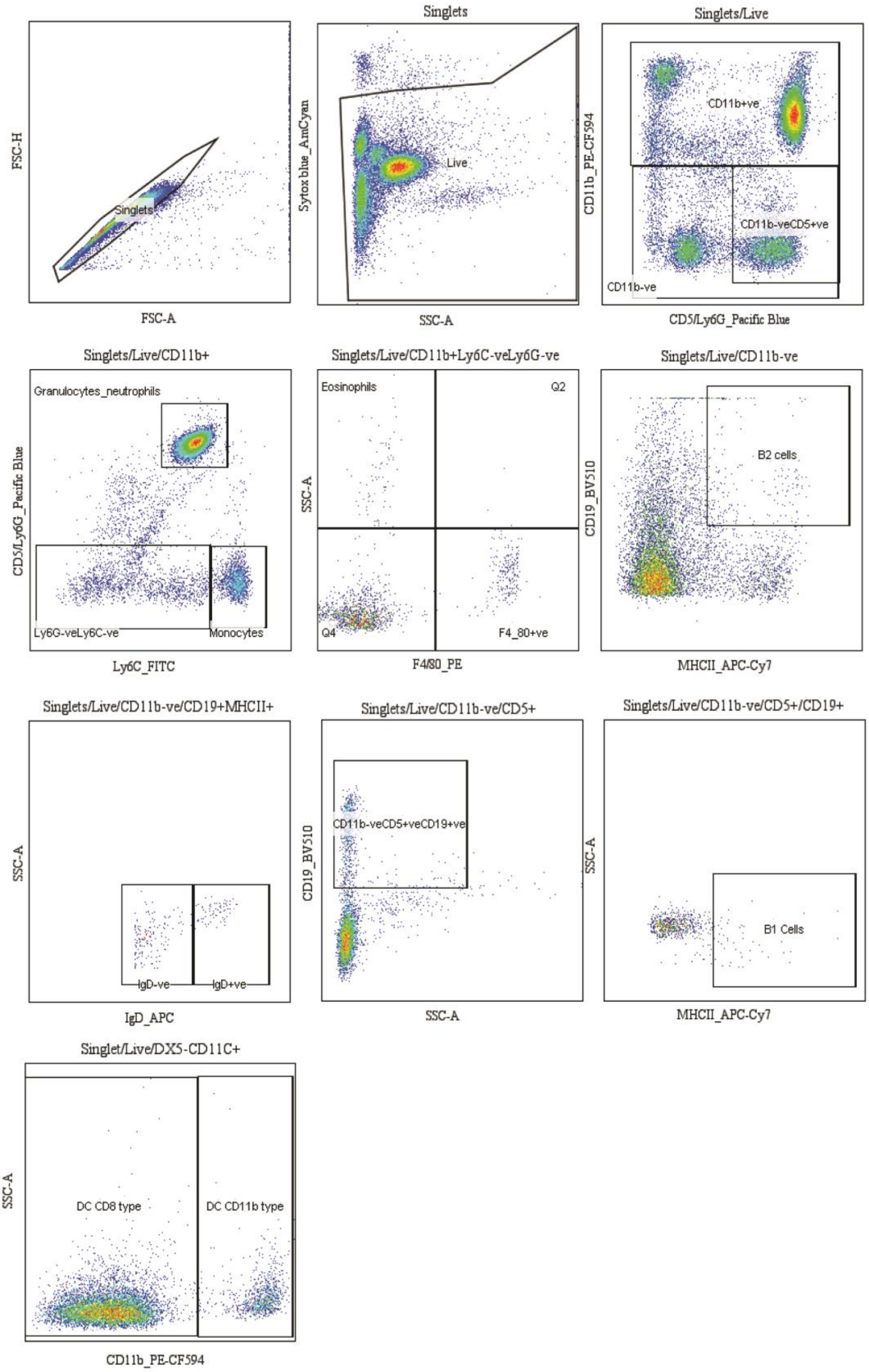

Fig S3: Flow cytometry panel 2 gating strategy.

Panel 1:				
**Marker / Antigen**	**Fluorophore**	**Concentration**	**Source**	**Cat no.**
Live/dead	SytoxBlue	1:10000	Invitrogen	S11348
CD5	BV421	1:200	BD biosciences	562739
CD4	FITC	1:800	BD biosciences	553047
CD8	PE-CF594	1:400	BD biosciences	562283
CD25	PECy7	1:400	BD biosciences	552880
CD62L	APC-Cy7	1:1600	BD biosciences	560514
CD44	PE	1:200	BD biosciences	553134
TCRd	BV510	1:100	BD biosciences	563218
DX5 (NK cell)	APC	1:200	BD biosciences	560628
**Panel 2:**				
**Marker / Antigen**	**Fluorophore**	**Concentration**	**Source**	**Cat no.**
Live/dead	SytoxBlue	1:10000	Invitrogen	S11348
F4/80	PE	1:400	BD biosciences	552957
CD19	BV510	1:400	BD biosciences	562956
DX5 (NK cell)	APC	1:200	BD biosciences	560628
Ly6C	FITC	1:400	BD biosciences	553104
Ly6G	BV421	1:400	BD biosciences	562737
CD5	BV421	1:200	BD biosciences	562739
CD11b	PE-CF594	1:4800	BD biosciences	562287
CD11c	PECy7	1:400	BD biosciences	558079
IgD	APC	1:200	BD biosciences	560868
MHCII (anti-Mouse I-A/I-E)	APC-Cy7	1:200	BioLegend	107628
**Panel 3:**				
**Marker / Antigen**	**Fluorophore**	**Concentration**	**Source**	**Cat no.**
CD4	FITC	1:800	BD biosciences	553047
FoxP3	PerCP-Cy5.5	1:200	BD biosciences	563902
CD25	PECy7	1:400	BD biosciences	552880

Table S1: Flow cytometry antibody and panels.

Gating strategy employed for classification of immune cells		
**Cells**	**Positive Markers**	**Negative markers**
Granulocytes/neutrophils	CD11b+Ly6G+Ly6C+	Sytox blue
Eosinophils	CD11b+SSC-Ahi	Sytox blue/Ly6C-Ly6G-
Macrophages	CD11b+SSClowF4/80+	Sytox blue/Ly6C-Ly6G-
Monocytes	CD11b+Ly6C+	Sytox blue/Ly6G-
Dendritic cells_CD8 type	CD11C+MHCII+	Sytox blue/DX5-CD11b-
Dendritic cells_CD11b type	CD11C+MHCII+CD11b+	Sytox blue/DX5-
Natural killer (NK) cells	DX5+	Sytox blue/CD5-
T helper (Th)	CD5+CD4+	Sytox blue/CD8-
T helper effector	CD5+CD4+CD44+	Sytox blue/CD8-
T regulatory effector	CD5+CD4+CD25+CD44+	Sytox blue/CD8-
T regulatory	CD5+CD4+CD25+	Sytox blue/CD8-
T cytotoxic (Tcyto)	CD5+CD8+	Sytox blue/CD4-
T cytotoxic effector	CD5+CD8+CD44+	Sytox blue/CD4-CD62L-
T cytotoxic naive	CD5+CD8+CD62L+	Sytox blue/CD4-CD44-
T cytotoxic resting	CD5+CD8+CD62L+CD44+	Sytox blue/CD4-
B2 mature cells	CD19+MHCII+IgD+	Sytox blue/CD11b-CD5-
B2 immature cells	CD19+MHCII+	Sytox blue/CD11b-CD5-IgD-
B1 cells	CD5+CD19+MHCII+	Sytox blue/CD11b-
NK-T cells	CD5+CD44+DX5+	Sytox blue
NK-T cell effector	CD5+CD44+DX5+	Sytox blue/CD62L-
NK-T cell resting	CD5+CD44+DX5+CD62L+	Sytox blue

Table S2: Flow cytometry gating strategy.
